# Molecular and Clinical Implications of Variant Repeats in Myotonic Dystrophy Type 1

**DOI:** 10.3390/ijms23010354

**Published:** 2021-12-29

**Authors:** Stojan Peric, Jovan Pesovic, Dusanka Savic-Pavicevic, Vidosava Rakocevic Stojanovic, Giovanni Meola

**Affiliations:** 1 Faculty of Medicine, Neurology Clinic, University Clinical Centre of Serbia, University of Belgrade, 11 000 Belgrade, Serbia; stojanperic@gmail.com (S.P.); vidosava_r@yahoo.co.uk (V.R.S.); 2 Center for Human Molecular Genetics, Faculty of Biology, University of Belgrade, 11 000 Belgrade, Serbia; jovan.pesovic@bio.bg.ac.rs; 3 Department of Biomedical Sciences for Health, University of Milan, 20100 Milan, Italy; giovanni.meola@unimi.it; 4 Department of Neurorehabilitation Sciences, Casa Di Cura del Policlinico, 20100 Milan, Italy

**Keywords:** myotonic dystrophy type 1, *DMPK* gene, repeat expansions, variant repeats, repeat interruptions, genetic modifier, phenotype variability, age at onset, somatic mosaicism

## Abstract

Myotonic dystrophy type 1 (DM1) is one of the most variable monogenic diseases at phenotypic, genetic, and epigenetic level. The disease is multi-systemic with the age at onset ranging from birth to late age. The underlying mutation is an unstable expansion of CTG repeats in the *DMPK* gene, varying in size from 50 to >1000 repeats. Generally, large expansions are associated with an earlier age at onset. Additionally, the most severe, congenital DM1 form is typically associated with local DNA methylation. Genetic variability of DM1 mutation is further increased by its structural variations due to presence of other repeats (e.g., CCG, CTC, CAG). These variant repeats or repeat interruptions seem to confer an additional level of epigenetic variability since local DNA methylation is frequently associated with variant CCG repeats independently of the expansion size. The effect of repeat interruptions on DM1 molecular pathogenesis is not investigated enough. Studies on patients indicate their stabilizing effect on *DMPK* expansions because no congenital cases were described in patients with repeat interruptions, and the age at onset is frequently later than expected. Here, we review the clinical relevance of repeat interruptions in DM1 and genetic and epigenetic characteristics of interrupted *DMPK* expansions based on patient studies.

## 1. Introduction

**Figure 1:**
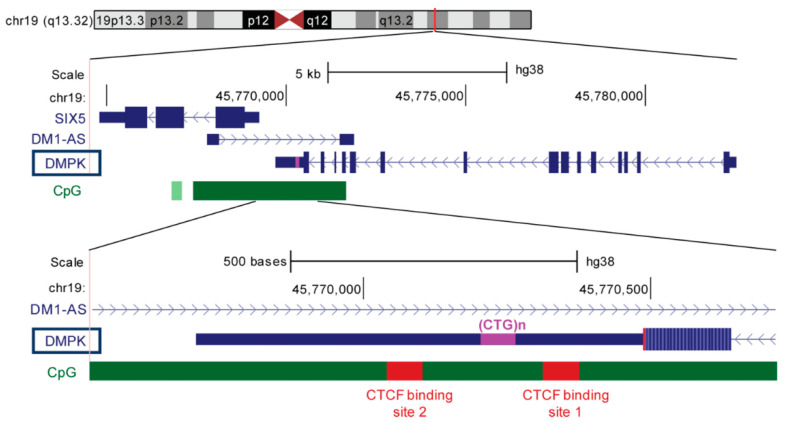
Annotation view of the 19q13.32 genomic region adapted from the UCSC genomic browser (https://genome.ucsc.edu/, accessed on 29 October 2021). CTG repeats (pink rectangle) are located in the 3′ untranslated region of the *DMPK* gene (exons—blue rectangles, introns—blue lines, 5′ and 3′ untranslated regions—thinner rectangles, direction of transcription—indicated by arrowheads on the introns). The CpG island (green rectangle) encompasses the 3′ end of the *DMPK* gene, the *DM1-AS* gene and the 5′ end of the *SIX5* gene. The CpG island contains two binding sites for the CTCF protein, one upstream (CTCF1) and one downstream (CTCF2) of the CTG repeats (their relative position is indicated with regard to CTG repeats).

Myotonic dystrophy (DM) is the most common form of muscular dystrophy in adults with a clinical prevalence of approximately 10 patients per 100,000 inhabitants [1]. Two forms have been described so far, myotonic dystrophy type 1 (DM1, OMIM#160900) and type 2 (DM2, OMIM#602668), which share a core clinical presentation (muscular dystrophy, myotonia, presenile cataracts, etc.), but are caused by mutations in two different genomic loci (reviewed in [2]). DM1 is caused by an expansion of CTG repeats in the 3′ untranslated region of the *DMPK* gene (Figure 1) (OMIM *605377) [3,4,5], while DM2 is caused by an expansion of CCTG repeats in the first intron of the *CNBP* gene (OMIM *116955) [6].

DM1 is an incurable, progressive, multi-system disorder that strongly impairs a patient’s quality of life and reduces life expectancy [7]. A recent screening of a population with diverse genetic ancestry estimated a genetic prevalence of 4.8 per 10,000 individuals [8], which makes DM1 one of the most common rare diseases. It is also the most variable repeat expansion disorder, and it is considered to be one of the most variable human disorders as well [1]. DM1 presents with the following symptoms: myotonia, muscle weakness and atrophy, cardiac conduction defects, cataracts, insulin resistance, endocrine, central nervous system, gastrointestinal and skin abnormalities, etc. This variability manifests as five clinical forms, mainly based on the age at disease onset: congenital (the most severe form), childhood, juvenile, adult, and late adult (the mildest form) [1,2,9,10]. Even more, each of these phenotypes is highly variable among individuals, including the presence/absence of certain symptoms, as well as their variable severity [9,11]. In addition, a prominent feature of DM1 is a genetic anticipation—an earlier age at onset and increased severity of the disease in subsequent generations of affected families [12,13]. The causes of DM1 clinical variability, which is reflected as a disease continuum rather than clearly distinct forms, have not been fully understood yet. The main driver is the underlying mutation, but other genetic, epigenetic, and environmental factors have been implicated [14].

The DM1-causing mutation widely varies in size, ranging from 50 to a few thousand repeats [15]. An inherently unstable nature of the (CTG)n array within the *DMPK* gene results in a huge genetic variability, not only among patients but also among tissues in individual patients. DM1 relevant tissues, such as skeletal muscle, heart, and cerebral cortex have a higher number of repeats than blood cells, among which the largest expansion was detected in the heart [16]. Depending on the initial expansion size, the number of repeats can change in each generation of affected families or upon each cell division, meaning that mutation rate of (CTG)n repeats within the *DMPK* locus can reach the value of 1 per generation or per cell division. Changes are biased towards further increases in expansion size [17,18,19]. This kind of genetic variability has a quantitative effect on the phenotype. Expansion size shows a positive correlation with the five DM1 clinical forms [9,20], and an inverse correlation with the age at disease onset [18]. Specific genotype-phenotype correlation together with expansion-biased instability of DM1 mutation in germ line cells is the molecular basis of genetic anticipation [3,20]. In addition, expansion-biased instability of DM1 mutation in somatic cells is continuous throughout a patient’s life [17,21], and is considered to contribute directly to the progressive nature of the disease [18]. A large CpG island with constitutively hypo- and hypermethylated regions overlaps with the 3′ part of the *DMPK* gene, encompassing the (CTG)n array as well (Figure 1) [22]. DNA hypermethylation, up- and downstream of very large expansions, is mostly seen in congenital DM1 patients [23,24]. Unlike other DM1 clinical forms which do not depend on the gender of the transmitting parent, the congenital form is almost exclusively maternally transmitted [20,25]. Recently, hypermethylation has been proposed as the molecular basis for maternal transmission of congenital DM1 form [23].

Although the expansion size plays a major role in determining DM1 phenotype, studies on patients have constantly shown an unexplained proportion of clinical variability. The mean expansion size is increasing with the increased severity of DM1 clinical forms, but there are large overlaps in the number of repeats among different forms [9,20,26]. It should be emphasized that somatic mosaicism of *DMPK* expansion imposes a difficulty for genotype-phenotype correlations [18]. Nevertheless, even when age at sampling and variability in somatic mosaicism are taken into account as confounding factors, the expansion size explains 65% of the variability in age at disease onset [18]. Additionally, local DNA hypermethylation can be seen in DM1 patients with clinical forms other than congenital [23,26,27,28]. About a decade ago, an additional source of genetic and epigenetic variability of DM1 mutation emerged as a factor that in cis modifies the effect of the expansion size. These are so-called repeat interruptions or variant repeats (e.g., CCG, CTC, CAG) in the expanded (CTG)n array of the *DMPK* gene. Discovering and proving the modifiers of rare diseases is not an easy task but repeat interruptions have drawn much attention due to different effects on phenotype, which have been described in several repeat expansions diseases, e.g., fragile X syndrome (FXS), a few types of spinocerebellar ataxias (SCA1, SCA2, SCA10, SCA17), and DM2 [29,30,31,32,33,34]. Early studies showing presence of interruptions in wild-type alleles, but not in expanded alleles, indicated their stabilizing role on repeat arrays. This is the case for DM2, in which the presence of several NCTG interruptions is limited only to wild-type alleles.

## 2. Variant Repeats in the (CTG)n Array of the *DMPK* Gene

Since its discovery in 1992, the (CTG)n array was considered to be uninterrupted or pure in both wild-type and expanded *DMPK* alleles. The only exception was an allele of 37 repeats that was found in an anonymous sperm donor and had the following structure (CTG)4(CCGCTG)16CTG [35]. Repeat interruptions in expanded (CTG)n array of the *DMPK* gene were described almost 15 years later [36,37]. It turned out that *DMPK* expansions are characterized by a high level of structural variability reflected in various types and patterns of repeat interruptions and their different locations within the (CTG)n array. All described interrupted *DMPK* expansions are unique for individual DM1 patients or families, as summarized in Table 1. Variant repeats such as CCG, CGG, CAG, and CTC have been identified at the 3′ end of *DMPK* expansions [36,37,38,39,40,41], and, more rarely, at the 5′ end [39,42]. The most commonly observed are CCG variant repeats. They are usually scattered as individual repeats, or make part of scattered or repeated CCGCTG hexamers, or are tandemly arranged as smaller or larger (CCG)n arrays. The occurrence of different types and patterns of variant repeats indicates that they probably originate by rare base substitutions within the pure (CTG)n array then spread along the expansion during processes involved in DNA metabolism. Rare findings of de novo occurring CTC [40] and CCG [43,44] variant repeats support this hypothesis of their origin.

The estimated frequency of interrupted *DMPK* expansions is 3–5% according to studies on DM1 patients from various populations [36,37,39,40,41]. This percentage could be even higher due to certain limitations of methods being used in patient screening. Repeat interruptions can be initially detected by repeat-primed PCR (RP-PCR) [45], which is today a routine method for molecular diagnosis of DM1 due to its simplicity and high specificity. Followed by additional rounds of RP-PCR with modified repeat-specific primer(s) and Sanger sequencing, the types and patterns of interruptions can be identified [37,40]. This is an important advantage compared to another method being used—*Aci*I digestion of small-pool PCR (SP-PCR) products, which is limited only to detection of variant CCG and CGG repeats [36]. In addition, the detection of a low number of these repeat interruptions at the very ends of *DMPK* expansion is biased and uncertain due to the low resolution of agarose gel used for resolving of *Aci*I digested SP-PCR products. On the other hand, *Aci*I digestion is beneficial for detecting CCG and CGG repeats which are located further from the expansion ends and/or are arranged in longer tandem arrays [40]. Although *Aci*I digestion was more commonly used for patient screening [36,43,46], it is of note that studies relying on RP-PCR also identified CCG repeat as the most common interruption [37,40]. Proof-of-concept studies have demonstrated the potential of PCR-based long-read PacBio sequencing technology to overcome the aforementioned limitations [43,44]. Harnessing long-read sequencing technologies will certainly bring a more complete picture of structural variations of not only *DMPK* expansions, but other expansions as well, including DM2.

## 3. Variant Repeats and DM1 Phenotype

Available literature data do not suggest a straightforward relationship between variant repeats and DM1 phenotype. However, several conclusions may be drawn. First, age at disease onset seems to be delayed in DM1 patients with variant repeats compared to DM1 patients without repeat interruptions, but with similar expansion size. Moreover, no congenital DM1 cases with variant repeats were described so far. Second, the phenotype in DM1 patients with interrupted *DMPK* expansions is usually reported as atypical, and it seems like these patients generally have a milder phenotype compared to DM1 patients with pure expansions. The detailed description of subjects with variant repeats in the (CTG)n array of the *DMPK* gene, reported in the literature so far, is presented in Table 1.

Age at disease onset is frequently reported as being later in DM1 patients with variant repeats compared to patients without interruptions [37,38,39,40]. Mathematical modeling showed a delay of 13.2 years in patients with repeat interruptions [47]. In another study, Miller and colleagues [48] compared patients with vs. without repeat interruptions matched for age, sex, and expansion size. Disease onset was delayed approximately seven years in those with repeat interruptions, but this was not of statistical significance. However, age at onset in DM1 patients with variant repeats may sometimes be as expected as early as or even earlier than in patients with pure CTG repeats [40]. Nevertheless, no congenital or childhood forms were reported in patients with repeat interruptions (Table 1) [36,37,42].

There is an important limitation in all studies that investigate age at onset as a measure. Age at disease onset is not a precisely defined category. Symptoms that may be considered as the first DM1 symptom may differ among different studies. Furthermore, due to the central nervous system involvement in DM1, it is expected that some patients cannot fully reconstruct when their symptoms appeared, or they neglect them due to their avoidant personalities [49,50]. Since patients with variant repeats have less cognitive impairment [38,40,41,51], they might better recognize and recall their first symptoms compared to those with pure (CTG)n array, which likely suggest an even higher difference in age at disease onset between these two groups. Aside from patients themselves, many physicians, including non-neuromuscular neurologists, are not always able to recognize early symptoms of DM1.

The majority of previous manuscripts reported that the phenotype of DM1 patients with variant repeats was milder, at least in some aspects of the disease, including less pronounced muscle weakness, less pronounced myotonia, and less pulmonary dysfunction (Table 1) [37,39,40,43]. Accordingly, in the OPTIMISTIC trial, DM1 patients with variant repeats had better mobility, ventilation, and cardiac status [52]. Additionally, the presence of repeat interruptions, together with *DMPK* expansion size, was a significant contributor to muscle strength in DM1 patients [46]. More precisely, patients with interrupted expansions were between healthy controls and DM1 patients with pure expansions in terms of their motor performance measured by Muscular Impairment Rating Scale, finger tapping, and grip strength [48].

The most consistent finding among different research groups is an absence or very mild brain involvement in DM1 patients with variant repeats, even at a later age (Table 1) [38,40,41,51]. Variant repeats (together with age at sampling and *DMPK* expansion size) were a significant explanatory variable of attention and executive function, apathy, fatigue, daytime sleepiness, and social behavior in DM1 patients [47]. Patients with repeat interruptions were between healthy controls and DM1 patients with pure expansions in terms of their cognitive (IQ, working memory, processing speed) and behavioral (depression, apathy, daytime sleepiness) performance [48]. However, there are no studies that assessed structural and micro-structural brain changes in DM1 patients with interruptions.

Interestingly, one patient with an interrupted expansion had testicular carcinoma [51]. DM1 patients carry a risk of different carcinomas [53], and based on the mentioned report, it seems that patients with variant repeats are not protected from this risk either.

It must be noted that many well-known DM1 symptoms in patients with repeat interruptions have been poorly reported in the literature. Based on the available results, while some symptoms were less pronounced in DM1 subjects with variant repeats, others were similar or even more severe compared to DM1 patients with the pure (CTG)n array. Particularly, some tissues may be less affected due to the presence of variant repeats (e.g., brain), while in some other tissues, their presence does not affect the phenotype or it makes it even more severe.

Although the clinical presentation of DM1 patients with variant repeats seems to be milder, they may worsen significantly when the disease is long-lasting. Santoro and colleagues [41] reported a patient with only 65 repeats and interruptions who developed a full multisystemic DM1 phenotype, 40 years after the disease onset. Similarly, Ballester-Lopez and co-authors [38] reported a family in which three subjects had first symptoms after the age of 50, but they developed a severe phenotype with limb and respiratory muscle weakness after the age of 60. It seems that the potential protective effect of interruptions on the DM1 phenotype may vanish with aging.

In addition to many papers where authors found milder clinical presentation in patients with variant repeats, Santoro and colleagues [41] concluded that there was no phenotypic difference in patients with vs. without variant repeats. A similar finding was reported for the Spanish family with variant repeats [38]. Furthermore, in some studies, subjects with variant repeats had an even more severe phenotype than expected. Musova and colleagues [37] reported a patient with only 37 repeats with (CCGCTG)13 hexamer interruption, who had myotonia since the age of seven (Table 1). One should consider the possibility that this patient could also have a chloride channel mutation as a concomitant disease as recently described [54]. Therefore, further genetic analysis of chloride and sodium channel mutations is needed to completely understand this unusual phenotype. A few additional individuals carrying *DMPK* alleles of a very similar size and a number of CCGCTG hexamers were reported. Two siblings were without DM1 symptoms [36], while one individual had a differential DM1 diagnosis that was excluded upon confirming the diagnosis of Prader–Willi syndrome [55] (Table 1).

**Table 1:** Genetic and phenotypic features of subjects with variant repeats in the *DMPK* (CTG)n array reported in the literature.

Subject	Sex	Transmission	Repeat Number	DM1 Allele Structure	Age at Onset	DM1 Clinical Symptoms	Symptoms Other Than DM1
**Cumming et al. 2021 [51]**							
Patient 1	M	paternal	NA	CCG at the 3′ end (exact structure not determined)	47 y	54 y—distal more than proximal MW, myotonia, cataract, no CI, 31 y—testicular carcinoma	/
Patient 2	F	paternal	270	CCG at the 3′ end (exact structure not determined)	NA	49 y—cataract, 57 y—no additional symptoms	/
**Fontana et al. 2020 [55]**							
I-2	F	NA	41	(CTG)6(**CCG**CTG)15(CTG)5	NA	/	84 y—senile cataract
II-1	M	maternal	41	(CTG)6(**CCG**CTG)15(CTG)5	asymp. at 54 y	/	/
III-1	F	paternal	41	(CTG)6(**CCG**CTG)15(CTG)5	NA	/	confirmed Prader–Willi syndrome—perinatal marked axial hypotonia, mild hypertonia in LE, facial weakness, respiratory failure, micro-retro-gnathia, later medium/severe CI
**Ballester-Lopez et al. 2020 [38]**							
Patient 1	F	NA	319	(CTG)n**CCG**(CTG)17**CCG**(CTG)29	52 y	72 y—mild generalized MW with severe axial weakness and dropped head, mild myotonia, cataract, pacemaker, NIV during night, no CI	/
Patient 2	F	NA	241	(CTG)n**CCG**(CTG)8(**CCG**)2(CTG)2 **CGG**(CTG)4**CCG**(CTG)30	50 y	62 y—mild myotonia, cataract, first-degree AV block, respiratory restriction, no CI	/
Patient 3	F	NA	368	(CTG)n(**CCG**CTG)3(CTG)3 (**CCG**CTG)3(CTG)3(**CCG**CTG)2 (CTG)17	in the 50 s	60 y—generalized weakness including axial, cataract, first-degree AV block, no CI, hypothyroidism	/
Patient 4	M	maternal	222	(CTG)n**CCG**(CTG)8(**CCG**)2(CTG)2 **CGG**(CTG)4**CCG**(CTG)30	asymp. at 35 y	/	/
Patient 5	F	maternal	547	(CTG)n(**CCG**CTG)3CTG(**CCG**)2 (**CCG**CTG)2(CTG)3(**CCG**CTG)2 (CTG)17	27 y	32 y—myotonia, cataract, first-degree AV block	/
**Cumming et al. 2018 [43]**							
DMGV14	F	paternal (de novo)	381	(CTG)180-240(**CCG**CTG)53-67 (CTG)53-67	asymp. at 33 y	/	hypothyroidism
DMGV182	M	paternal (de novo)	293	(CTG)200-300**CCG**(CTG)41-59	NA	43 y—mild masseter myotonia, early cataract, dermal fibrosis	/
DMGV15	F	paternal (de novo)	327	(CTG)260-320(**CCG**CTGCTG)10-14 (CTG)15-23	asymp. at 46 y	/	mitral valve replacement for congenital heart anomaly
**Tome et al. 2018 [42]**							
A1	F	NA	170	(CTG)31**CAG**(CTG)n	NA	69 y—severe MW, nasogastric enteral feeding, died at 72 y	/
A2	F	maternal	150	(CTG)31**CAG**(CTG)n	NA	49 y—severe MW, myotonia, heart conductive defect, ICD implanted, died of heart attack at 58 y	/
A3	F	maternal	140	(CTG)29**CAG**(CTG)n	asymp. at 32 y	/	/
A4.1	F	maternal	125	(CTG)31**CAG**(CTG)n	asymp. at 13 y	/	/
A4.2	F	maternal	130	(CTG)31**CAG**(CTG)n	asymp. at 7 y	/	/
A4.3	NA	maternal	125	(CTG)30**CAG**(CTG)n	/	therapeutic abortion	/
B1	F	NA	365	(CTG)11**CCG**(CTG)2**CCG** (CTG)4**CCG**(CTG)n	NA	58 y—severe MW, nasogastric enteral feeding	/
B2	F	maternal	310	(CTG)11**CCG**(CTG)2**CCG** (CTG)4**CCG**(CTG)n	NA	33 y—no MW, myotonia	/
B3.1	NA	maternal	300	(CTG)11**CCG**(CTG)2**CCG** (CTG)4**CCG**(CTG)n	/	therapeutic abortion	/
B3.2	NA	maternal	235	(CTG)11**CCG**(CTG)2**CCG** (CTG)4**CCG**(CTG)n	/	therapeutic abortion	/
B3.3	NA	maternal	250	(CTG)11**CCG**(CTG)2**CCG** (CTG)4**CCG**(CTG)n	/	therapeutic abortion	/
**Pesovic et al. 2017 [40], Pesovic et al. 2018 [56]**							
DF1-1	F	NA	520	(CTG)n(**CCG**CTG)3(CTG)4 (**CCG**CTG)2CTG**CCG**(CTG)17	39 y	57 y—proximal and distal MW, myotonia, cataract, sinus bradycardia, mitral regurgitation, glucose intolerance	/
DF1-2	M	maternal	350	(CTG)n(**CCG**CTG)3(CTG)4 (**CCG**CTG)2CTG**CCG**(CTG)17	30 y	37 y—distal MW, calf hypertrophy, myotonia, no CI	/
DF1-3	M	maternal	450	(CTG)n(**CCG**CTG)3(CTG)4 (**CCG**CTG)2CTG**CCG**(CTG)17	15 y	30 y—distal MW, calf hypertrophy, myotonia, no CI, infertility	/
DF2-1	M	NA	320	(CTG)n(**CCG**)36(CTG)n **CCG**(CTG)7**CCG**(CTG)12	40 y	45 y—proximal and distal MW, myotonia, winging scapula, cataract, borderline PR interval, prolonged LV relaxation, no CI	/
DF2-2	F	paternal	200	(CTG)n(**CCG**)40(CTG)24 **CCG**(CTG)7**CCG**(CTG)12	12 y	14 y—myotonia	/
DF3-1	F	NA	240	(CTG)n(**CCG**)3(CTG)6(**CCG**)3 (CTG)7**CCG**(CTG)8**CCG**(CTG)8	45 y	46 y—proximal MW, myotonia, cataract, prolonged LV relaxation, hypothyroidism	/
DF3-2	F	maternal	187	(CTG)n(**CCG**)3(CTG)6(**CCG**)3 (CTG)7**CCG**(CTG)8**CCG**(CTG)8	31 y	31 y—myotonia, leg pain	/
DF4-1	F	maternal	300	(CTG)n**CCG**(**CCG**CTG)4CTG(**CCG**CTG)4CTG(**CCG**CTG)2(CTG)5 (**CCG**)4(CTG)6(**CCG**)3(CTG)20	39 y	59 y—proximal and distal MW, myotonia, cataract, borderline LV hypertrophy, mild restriction, no CI, hyperthyroidism, hyperparathyroidism	/
DF5-2	F	paternal (de novo)	250	(CTG)n**CTC**(CTG)26	22 y	27 y—distal MW, myotonia, cataract, borderline PR interval, no CI	/
**Botta et al. 2017 [39]**							
A1	M	NA	1000–1400	(CTG)880-1280(CTG)2 **CCG**(CTG)112**CCG**(CTG)4	58 y	66 y—MW	/
A2	F	paternal	475–640	(CTG)437-602(CTG)14 **CCG**(CTG)18**CCG**(CTG)4	31 y	myotonia	/
A3	NA	maternal	500	(CTG)380(CTG)28**CCG**(CTG)40**CTC** (CTG)36**CCG**(CTG)8**CCG**(CTG)4	/	prenatal sample	/
B1	F	NA	740–930	(CTG)699-889(**CCG**CTG)2 (**CCG**)2(CTG)3(**CCG**CTG)3(CTG)26	51 y	55 y—MW, myotonia, cataract	/
B2	F	maternal	450–550	(CTG)372-472(CTG)16**CCG** (CTG)2(**CCG**CTG)4CTG (**CCG**CTG)4**CCG**(**CCG**CTG)4 **CCG**(**CCG**CTG)5(CTG)22	asymp. at 28 y	/	/
C1	F	NA	140	(CTG)30(**CCG**)2 (CTG)2**CCG**(CTG)105	58 y	myotonia	/
C2	F	maternal	121	(CTG)28(**CCG**)2 (CTG)2**CCG**(CTG)88	37 y	40 y—myotonia	/
C3	NA	maternal	113	(CTG)31(**CCG**)2(CTG)2 **CCG**(CTG)13**CCG**(CTG)63	/	prenatal sample	/
D	F	NA	600–700	(CTG)514-614(CTG)68 (**CCG**)9(CTG)9	35 y	38 y—myotonia	/
E	F	NA	500–660	(CTG)404-564(CTG)33 (**CCG**CTG)28(CTG)7	49 y	56 y—myotonia	/
F	M	NA	250	(CTG)208(CTG)5 (**CCG**CTG)16(CTG)5	66 y	70 y—mild myotonia	/
G	M	NA	400–580	(CTG)330-510(CTG)8 (**CCG**CTG)17(CTG)2**CCG**(CTG)25	61 y	71 y—MW, myotonia	/
H	M	NA	175	(CTG)133(CTG)8**CCG**(CTG)5 (**CCG**)2CTG(**CCG**)4(CTG)2(**CCG**)4 CTG(**CCG**)2(CTG)2**CCG**(CTG)9	46 y	49 y—MW, myotonia	/
I	M	NA	265–772	(CTG)188-650CTG**CCG** (CTG)2**CCG**(CTG)7**CCG**(CTG)28 **CCG**(CTG)7**CCG**(CTG)22	15 y	20 y—myotonia, cataract	/
**Santoro et al. 2013 [41]**							
pt1	NA	paternal	550–700	(CTG)n(**CCG**CTGCTG)46(CTG)5	NA	late-onset DM1 with distal MW, myotonia, cataract, sinus bradycardia, RBBB, occasional ectopic premature complexes	/
pt2	NA	paternal	600–830	(CTG)n(**CCG**CTGCTG)61(CTG)5	NA	late onset proximal MW, myotonia, cataract, pacemaker implanted due to long HV, later ICD implanted due to non-sustained ventricular tachycardia, hypothyroidism	/
pt3	NA	NA	65	(CTG)n(**CCG**CTGCTG)5(CTG)3	30 y	70 y—distal MW in UE, and proximal MW in LE, cataract, previous heart attack, first-degree AV block, RBBB, mild dysexecutive syndrome, diabetes, hypothyroidism	/
pt4	NA	NA	900	(CTG)n(CTG)8**CCG**(CTG)2**CCG** (CTG)2**CCG**(CTG)5**CCG** (CTG)8**CCG**(CTG)4**CCG**(CTG)16	28 y	32 y—facial and hand MW, first-degree AV block, prolonged HV, pacemaker and later ICD implanted	/
pt5	NA	maternal	970	(CTG)n(CTG)5**CCG**(CTG)5 **CCG**(CTG)6**CCG**(CTG)12	20 y	78 y—myotonia, cataract, first-degree AV block, mild respiratory restriction, NIV during sleep	/
**Braida et al. 2010 [36]**							
family co-segregating DM1 with CMT	/	/	170–225	(CTG)n(**GGC**)3G(**CCG**)20 (**CCG**CTG)14(CTG)35	/	typical DM1	Charcot–Marie–Tooth and/or acute encephalopathy attacks and/or early hearing loss
**Musova et al. 2009 [37]**							
A-1	NA	maternal	230	(CTG)n**CTC**(CTG)9(**CCG**CTG)2 (CTG)2**CCG**(CTG)5**CCG**(CTG)13	/	prenatal sample	/
A-2	F	paternal	300	(CTG)n**CTC**(CTG)9(**CCG**CTG)2 (CTG)2**CCG**(CTG)5**CCG**(CTG)13	asymp. at 31 y	/	/
A-3	F	paternal	400–500	(CTG)n**CTC**(CTG)7**CCG**(CTG)5 **CCG**(CTG)5**CCG**(CTG)13	asymp. at 23 y	/	/
A-4	M	NA	600–800	(CTG)n**CTC**(CTG)9**CCG**(CTG)5 **CCG**(CTG)5**CCG**(CTG)13	40 y	distal MW, myotonia, cataract, hyperglycemia, axonal polyneuropathy	/
A-5	F	NA	450–650	(CTG)n**CTC**(CTG)9 (**CCG**CTGCTG)5**CCG**(CTG)10	NA	mild DM1, 42 y—fatigue, first-degree AV block	/
A-6	F	maternal	650–750	(CTG)n**CTC**(CTG)9 (**CCG**CTGCTG)4**CCG**(CTG)10	asymp. at 29 y	/	/
A-7	M	maternal	270	(CTG)n**CTC**(CTG)9 (**CCG**CTGCTG)5**CCG**(CTG)10	asymp. at 31 y	/	/
B-1	M	NA	450	(CTG)n(**CCG**CTG)33-39 **CCG**(**CCG**CTG)3(CTG)18	NA	mild DM1, 40 y—cataract, 50 y—masticatory and hand myotonia, cramps	/
B-2	M	paternal	400	(CTG)n(**CCG**CTG)35-37 (**CCG**)12CTG**CCG**(CTG)11	asymp. at 25 y	/	/
C	F	NA	700	(CTG)n(**CCG**CTG)2(**CCG**)8CTG (**CCG**)6CTG(**CCG**)6CTG**CCG**CTG (**CCG**)2CTG(**CCG**CTG)3(**CCG**)2 CTG(**CCG**CTG)3CTG(**CCG**CTG)4 CTG(**CCG**CTG)4**CCG**(**CCG**CTG)3 (CTG)3(**CCG**CTG)2(CTG)10	NA	23 y—cramps, 41 y—distal MW, myotonia	/
D	M	NA	37	(CTG)6(**CCG**CTG)13(CTG)5	/	/	congenital myotonia-like symptoms since age of 7, mild muscle hypertrophy
E-1	M	paternal	43	(CTG)6(**CCG**CTG)16(CTG)5	/	/	since birth muscle stiffness, 20 y—short stature, dysmorphic features, contractures, distal leg muscle atrophy, hypertrophic cardiomyopathy
E-2	M	NA	43	(CTG)6(**CCG**CTG)16(CTG)5	/	/	short stature, contractures

M—male, F—female, y—years old, MW—muscle weakness, CI—cognitive impairment, UE—upper extremeties, LE—lower extremities, NIV—non-invasive ventilation, AV—atrioventricular, LV—left ventricular, RBBB—right bundle branch block, ICD—implantable cardioverter defibrillator, NA—not available.

Some authors reported unusual phenotypes in DM1 patients with interrupted expansions (Table 1). Musova and co-authors [37] reported a phenotype of myotonia in a patient mentioned above. On the other hand, Pesovic and colleagues [40] reported patients with proximal weakness, calf hypertrophy, and absent percussion myotonia, resembling myotonic dystrophy type 2 rather than type 1. Accordingly, in two out of five Italian patients with repeat interruptions, proximal muscle weakness was reported as a presenting symptom of the disease which is more typical for myotonic dystrophy type 2 than 1 [41]. Furthermore, the Spanish group reported severe axial weakness in their two patients with variant repeats from the same family, with one patient having even dropped head, which is completely unexpected in DM1 [38].

In addition, very complex phenotypes of patients with interruptions were described by several research groups (Table 1). A Dutch family was characterized by DM1 associated with Charcot–Marie–Tooth disease with or without early hearing loss and recurrent encephalopathic attacks [36]. Czech authors found a patient with short stature, foot and facial deformities, ptosis, mild contractures, peroneal atrophy, and hypertrophic cardiomyopathy, without myopathic pattern and myotonia on needle electromyography [37]. His father and grandfather also had contractures and short stature. Intriguingly, the Czech patients had only 43 repeats interrupted by CCGCTG hexamers, which is hard to associate with such a severe and unusual phenotype. Cumming and co-authors [43] described a patient with an interrupted expansion with a progenitor allele of 303 repeats and modal allele of 379 repeats. Interestingly, the patient did not have any muscle symptoms at age of 46, while she had mitral valve replacement for congenital heart anomaly that is unusual for DM1. These complex phenotypes are unexpected in DM1, thus further genetic analysis should be performed in order to exclude other genetic, concomitant diseases.

It seems hard to explain such a huge phenotypic variability in DM1 subjects with variant repeats, but it can probably be expected due to a pronounced structural variability of *DMPK* expansions, each being unique concerning: type of interruptions (CCG as the most common, but also others—GGC, CTC, CAG), pattern (simple or complex), length of interruptions compared to the full expansion length, and their location within the expansion.

## 4. Molecular Effects of Interrupted (CTG)n Array in the *DMPK* Locus

Based on more stable intergenerational transmission of interrupted vs. pure *DMPK* expansions and a less pronounced somatic mosaicism in DM1 patients, variant repeats have been recognized as a *cis*-factor, having a stabilizing role on *DMPK* expansions in both germ line and somatic cells [36,40,42,56]. This molecular effect is believed to mediate the absence of congenital cases and delay the age at disease onset in DM1 patients with variant repeats. Several studies described altered DNA methylation levels in the regions surrounding interrupted *DMPK* expansions [27,28,57], raising a question of an association between repeat interruptions and epigenetic modifiers of DM1 phenotype. More downstream molecular effects of variant repeats in the *DMPK* locus are not explored enough. Examination of muscle biopsies from DM1 patients showed that variant CCG repeats do not affect the formation of toxic RNA foci and their co-localization with MBNL proteins [41], which is the key mechanism of molecular pathogenesis in DM1 (reviewed in [58]). This is in line with the computational predictions showing that various patterns of CTG and CCG repeats form similar RNA secondary structures, without effect on the binding of MBNL proteins [36]. Nonetheless, it is still not known if RNA molecules carrying a large number and/or different patterns of repeat interruptions could interact with additional RNA-binding proteins, which could increase the complexity of disturbed RNA metabolism. A possible effect of variant repeats on RAN translation, an additional mechanism of molecular pathogenesis being implicated in DM1 [59], has not been examined so far. The group of prof. Ranum recently showed that variant CCG•CGG repeats in bidirectionally transcribed (CTG)n array associated with SCA8 increase stability of RNA hairpins and affect RAN translation by increasing toxicity of polyGln polypeptides interrupted by Arg, as well as increasing steady-state level of polyAla and polySer polypeptides [60]. These molecular effects may explain a higher penetrance of interrupted SCA8 expansions in comparison to the pure ones, and the observed inverse correlation of age at onset with the number of variant CCG•CGG repeats, but not with SCA8 expansion size [60].

### 4.1. Variant Repeats and Somatic Instability of DMPK Expansions

Studies on DM1 patients have constantly shown that repeat interruptions commonly confer stability to *DMPK* expansions in somatic cells regardless of the interruption type, pattern, and location, or whether they are inherited or arising de novo [36,42,43,56]. Moreover, even one variant repeat, at either 5′ or 3′ end, increases somatic stability of *DMPK* expansions [42,56]. Importantly, one study showed that the stabilizing effect of variant repeats on *DMPK* expansions in somatic cells can mediate the observed delayed age at disease onset in DM1 patients with vs. without repeat interruptions [56].

The greater stability of interrupted compared to pure expansions was demonstrated using different approaches to control factors contributing to the variability of somatic mosaicism among DM1 patients [18]. One of them involved comparisons of SP-PCR profiles which indicated a visually lesser degree of somatic mosaicism in patients carrying interruptions in comparison to a few patients with pure expansions, matched on age at sampling and expansion size [42]. Another study implemented the same approach, but instead of visual comparisons, a difference between the modal and the progenitor expansion size, determined by SP-PCR, was used as a simple measure of somatic instability [43]. However, such comparisons of DM1 patients are limited by the fact they do not account for individual-specific factors contributing to the variability in somatic mosaicism. According to the landmark study on a large cohort of DM1 patients, approximately 90% of the variability in somatic mosaicism could be accounted for by the expansion size and the age at patient’s sampling, while the remaining 10% of the unexplained portion of variability could be attributed to individual-specific modifying factors [18]. Importantly, the study employed the single-molecule SP-PCR which provides the most accurate measurement of the somatic mosaicism in DM1 patients [61]. To control for the overall variability in somatic instability of *DMPK* expansions (including individual-specific factors), two studies examined data on DM1 patients with variant repeats together with data on a large group of DM1 patients with pure repeats [36,56]. Both studies showed that the level of somatic instability in DM1 patients with interrupted vs. pure expansions was statistically significantly lower than expected based on mathematical models inferred from the patients with pure expansions [18]. The study from Braida and co-workers [36] left an open question whether the obtained result was driven solely by the specific structure of interrupted *DMPK* expansions or by shared individual-specific factors given that they analyzed patients from a single family. On the other hand, Pesovic and co-authors [56] compared a group of unrelated patients with interrupted expansions of various structures, thus providing a more reliable result about the overall stabilizing role of variant repeats on *DMPK* expansions in somatic cells.

Analysis of other aspects of somatic mosaicism in DM1 patients with variant repeats comes from the research on buccal cells, as well as blood cells over time in a few patients [56]. Results indicated that instability of variant *DMPK* expansions is tissue-specific, like in pure *DMPK* expansions. A shift in expansion size frequency distribution towards larger alleles was noticed. During the follow-up for a relatively short period, some patients were characterized by an increase in modal expansion size, while others showed no change in their expansion size frequency distributions. We are referring readers to Figure 4 (showing somatic instability of *DMPK* expansions in blood cells over time) and Table 1 (showing the appropriate structure of *DMPK* expansions in analyzed patients) in Pesovic et al. [56]. In addition, the structures of repeat interruptions over time were stable in all examined patients. Furthermore, DM1 patients with interrupted vs. pure expansions had a significantly smaller increase in modal expansion size over time, based on the mathematical model of Martorell and co-authors [56,62]. Therefore, these results point out that the main feature of interrupted *DMPK* expansions is greater stability when compared to pure expansions.

An ongoing somatic instability biased towards further expansions is of particular clinical interest as it was proposed to be the molecular basis for the progressive nature of DM1 symptoms, i.e., variability in somatic mutation dynamics among patients is a source of variation in disease severity [18]. This assumption has been recently confirmed for age at disease onset in a longitudinal study showing that individual-specific variation in the increase of modal expansion size over time (not explained by the inherited expansion size, age at sampling and time interval) was inversely associated with individual-specific variation in age at onset (not accounted for by the inherited expansion size) [19]. In line with this, the results of Pesovic and co-authors [56] showed that individual-specific variation (not explained by the inherited expansion size and age at sampling) has a greater influence on age at disease onset in DM1 patients with interrupted vs. pure expansions. Therefore, repeat interruptions seem to be of clinical importance for disease course, at least by delaying the age at disease onset. The most likely underlying molecular mechanism is stabilizing effect of variant repeats on *DMPK* expansions in somatic cells. The role of somatic instability and repeat interruptions in modifying age at disease onset has been suggested in Huntington’s disease as well. Increased somatic instability, due to loss of CAA variant repeat in the (CAG)n array associated with Huntington’s disease, has been proposed as the most probable cause of an increased penetrance of alleles in the ‘gray’ zone and an earlier age at disease onset (reviewed in [63]).

Analyzing a larger group of unrelated DM1 patients with variant repeats is needed to further differentiate effects of types, patterns, and location of repeat interruptions on somatic instability and age at disease onset. Studies should be preferentially done by the approaches that take into account all factors influencing the variability of somatic mosaicism and age at onset [36,56].

### 4.2. Variant Repeats and Germ-Line Instability of DMPK Expansions

Both expansion and contraction events are possible during intergenerational transmission of *DMPK* expansions with variant repeats in DM1 families. However, contractions or, at least, stable transmissions are more likely, both in paternal and maternal transmissions [36,37,38,40,44]. For examples of maternal and paternal transmission of interrupted *DMPK* expansions we are referring readers to Figures 3 and 4 (subjects DF1-1, DF1-2, DF1-3, DF2-1 and DF2-2) in Pesovic et al. [40]. More precisely, the frequency of contractions or stable transmissions is significantly higher in DM1 families carrying interrupted vs. pure expansions (68.4 vs. 6.4%), while the frequency of expansions is lower (31.6% vs. 93.6%) [40,64]. This is a striking deviation from the expected behavior of the *DMPK* expansion upon transmission, in terms of both the direction of the expansion size change and the sex of the transmitting parent [20,64,65,66]. In a general DM1 population *DMPK* expansions of 50–100 repeats are usually stable in maternal transmissions and commonly undergo expansions in paternal transmissions [67]. On the other hand, *DMPK* expansions of >100–200 repeats are inherited unstably by both sexes. In this size range, transmissions mostly result in expansions, but stable transmissions and contractions can occur, particularly in paternal transmissions [64,66]. Interestingly, even a *DMPK* expansion carrying a single CAG interruption was characterized by stable transmission or contraction in successive generations [42], suggesting that a single base substitution within CTG repeat may be sufficient to increase the meiotic stability of *DMPK* expansion. Although transmissions of interrupted *DMPK* expansions showed no specificity related to the sex of the transmitting parent [40], it is worth mentioning that all de novo occurring variant *DMPK* expansions, described so far, were transmitted by fathers, whether it was an expansion carrying a single CTC repeat [40], or different patterns of CCG repeats [43,44].

The meiotic stability of variant *DMPK* expansions is considered to be an explanation for the absence of congenital form in DM1 families reported so far [36,39,42]. Congenital cases were absent even in a large family with several transmissions by adult-onset females [36]. However, a recent finding speaks against the claims that congenital DM1 form is highly unexpected in patients with repeat interruptions [43]. From eight separate in vitro fertilizations performed in a female patient with an interrupted expansion of 418 repeats in size, one embryo had approximately the same expansion size as the mother, while the remaining seven had substantially longer expansions, including one with over 1300 repeats [43]. Altogether, data collected so far support repeat interruptions as *cis*-factors that stabilize *DMPK* expansions in germ line cells and modify not only the effect of expansion size but also the parent-of-origin effect on intergenerational transmissions.

In addition to DM1, variant repeats were shown to stabilize intergenerational transmissions of repeats in other expansion disorders, such as FXS [33], SCA10, and SCA17 [30,32]. A stabilizing effect is seen on wild-type alleles, premutation alleles, and full mutations depending on the type of disease.

Unfortunately, in DM1 families carrying repeat interruptions, some intergenerational contractions were accompanied by an earlier age at disease onset, suggesting unexpected genetic anticipation [39,40]. Similar findings were reported in patients with SCA10, in whom, in almost all cases, a contraction of variant expansions was accompanied by an earlier onset of disease symptoms [32]. It is of note that an early finding in a large DM1 patient group showed that almost half of the cases of intergenerational contractions were accompanied by genetic anticipation [64]. There was an excess of paternally transmitted contractions and they were less frequently accompanied by worsening clinical presentation [64]. However, one should be aware of possible bias in the ascertainment of age at disease onset in offspring and be careful about the interpretation of the intergenerational mutational dynamics. Intergenerational change in expansion size is the result of meiotic instability in germ cells of the transmitting parent and mitotic instability in blood cells of the parent and the offspring. It is challenging to unambiguously determine the inherited expansion size in child, thus the question remains whether the abovementioned cases are indeed contractions, followed by an earlier age at onset, or pseudocontractions in which there is, in fact, an intergenerational increase in repeat number, masked by somatic instability in parents.

### 4.3. Variant Repeats and DNA Methylation in the DMPK Locus

Molecular effects of repeat interruptions in DM1 seem to be more complex since they not only increase genetic variability of *DMPK* expansions, but also epigenetic variability of the *DMPK* locus. Notably, pieces of evidence suggesting the clinical relevance of epivariations associated with *DMPK* expansions are emerging [23,57]. The (CTG)n array is embedded within a large CpG island of 3.5 kb in size, which encompasses a complex genomic region covering the 3′ end of the *DMPK* gene, the *DM1-AS* gene, and the 5′ end of the downstream *SIX5* gene (Figure 1). In non-DM1 individuals, this island is characterized by a pattern of constitutive hyper- and hypomethylation of certain CpG sites [22]. The CpG island also includes two CTCF-binding sites near the CTG repeats, one located upstream and the other one downstream (Figure 1), both of which remain unmethylated in unaffected individuals [68].

The hypermethylation of CpG sites upstream of the (CTG)n array was first observed more than 20 years ago in the congenital form of the disease [24]. It was associated with large expansions in these patients and believed to be driven by expansion itself in a polarized manner, spanning only upstream regions [69,70]. However, more recent findings have suggested hypermethylation at both CTCF sites (upstream and downstream of expansion) is almost exclusive in congenital patients, thus being proposed as a clear biomarker for this DM1 form, in addition to expansion size [23,26]. Furthermore, increased methylation is anticipated to be the underlying mechanism for the parent-of-origin effect for the maternal-biased transmission of the congenital form [23]. However, rare paternal transmissions of hypermethylated expansions associated with congenital DM1 have been only recently observed [26]. Hypermethylation of the CpG sites surrounding *DMPK* expansion has been reported in non-congenital DM1 forms as well [23,26,27,28], although more rarely compared to congenital form. Moreover, the methylation levels in expanded *DMPK* locus were shown to be stable over time in blood cells [27].

The discovery that hypermethylation can be associated with interrupted *DMPK* expansions is quite intriguing because patients with variant repeats frequently have milder symptoms than expected, thus being situated on the opposite side of the DM1 phenotypic spectrum compared to the most severe congenital form. Santoro and co-authors [28] first described hypermethylation of the downstream CpG sites in the majority of patients with variant expansions, while the upstream CpGs remained unmethylated. Hypermethylation in the downstream region was observed in patients having more abundant CCG interruptions along the (CTG)n array independently of the expansion size [28]. This polarized pattern of methylation has been recently confirmed in two additional studies, although data on the expansion size and structure were unknown [27,57]. Our unpublished results support previous findings on downstream hypermethylation in several patients with various patterns of CCG variant repeats at the 3′ end of expansion with different sizes. In line with the previous finding [28], we observed a higher level of methylation in those patients carrying more complex patterns of CCG interruptions. In several of our families, sampled at two different time points, the methylation levels were stable over time (our unpublished data). Interestingly, methylation of the surrounding CpG sites was neither detected in patients with a single CAG repeat or several CCG repeats at the 5′ end of relatively small *DMPK* expansions [42], nor in our patient with one CTC repeat at the 3′ end of *DMPK* expansion. Taken together, these results suggested that the degree of methylation in the region downstream of variant expansions depends more on the type and pattern of interruptions than on the expansion size.

The association of downstream DNA methylation with the presence of CCG repeats in *DMPK* expansions is noteworthy due to known DNA methylation patterns in other diseases caused by GC-rich expansions. Expansions of CGG repeats in the *FMR1* gene larger than 200 repeats are known to be methylated in the majority of patients with FXS [71]. In the case of *FMR1* expansions, the methylation originates upstream and extends into a promoter sequence and down to the CGG repeats [72]. More recently, methylation was detected at the *C9orf72* locus in which expansions of G4C2 hexamers are the underlying cause of the most common inherited form of amyotrophic lateral sclerosis/frontotemporal dementia [73]. Here, methylation is concentrated on G4C2 repeats at the 5′ end of expansion, involving upstream CpG sites as well [73]. Methylation patterns in these repeat-expansion disorders indicated DNA methylation could be a consequence of the presence of a large number of GC-rich repeat motifs within the repeated tract, with some locus-specific differences in the location of initiation and spreading. This association has been recently confirmed in a large study on epivariations in more than 23,000 human genomes which identified 25 genomic loci where hypermethylation was associated with unstable expansions of CGG repeats, including those whose pathological significance is not known yet [74].

Whether epivariations associated with interrupted *DMPK* expansions mediate DM1 phenotype variability remains unclear. Legare and colleagues [57] reported on the contribution of DNA methylation downstream of the (CTG)n array, independently of the expansion size, to the variability of muscular and respiratory phenotypes. The study included only adult DM1 patients among whom 8.9% had interrupted expansions which were associated with a higher DNA methylation level downstream of the (CTG)n array. However, the authors did not comment on whether the contribution of DNA methylation to the variability of muscle and respiratory phenotypes was driven by patients with variant repeats.

The question regarding the mechanism(s) by which DNA methylation could affect the phenotype is still without an answer. We tend to believe DNA methylation could, at least in part, be responsible for the stabilizing effect of repeat interruptions on *DMPK* expansions. Indeed, increased DNA methylation downstream of the (CTG)n array was associated with lower somatic instability [57], while local hypermethylation is known to be associated with increased stability of full mutation associated with FXS [75]. To test this hypothesis, further research is required on cell model systems that would enable separate examination of the effects of variant repeats and DNA methylation.

## 5. Future Perspectives and Conclusions

About a decade-long research on variant repeats in DM1 patients has broadened our perception of genetic and epigenetic variability of expanded *DMPK* locus and its relation to phenotype variability of DM1. Lessons we have learned have important implications of clinical relevance with regard to genetic diagnostics, genetic counseling, and stratifying patients for clinical trials. Considering that the structure of interrupted *DMPK* expansions is unique to individual patients, a false-negative RP-PCR result is possible. To increase the reliability and accuracy of RP-PCR, which is routinely used for molecular diagnostics in a clinical setting, it has been suggested to perform bidirectional RP-PCR (one reaction targeting the 5′ end of the (CTG)n array and another targeting the 3′ end) [76]. Genetic counseling for DM1 is very complex due to extremely variable clinical presentation. In addition, due to a broad genotype-phenotype correlation, the expansion size has no predictive value in clinical practice [15]. The fact that repeat interruptions could affect the mutational dynamics across generations with direct phenotypic consequences poses a new challenge in providing adequate genetic advice to DM1 families with variant expansions. Screening for variant repeats should be performed for clinical trial purposes since the presence of repeat interruptions needs to be included as a factor in the randomization process. In the OPTIMISTIC study, the percentage of patients with variant repeats was higher than in the general DM1 population (8.4%), which suggests that variant repeat carriers were over-recruited, probably due to their preserved cognition and higher motivation to take part in the trial [47]. This may also be the case in future trials.

Nevertheless, acquired knowledge seems to be only a tip of the iceberg. Many questions have only been raised and future research should overcome limitations of current methods and study design. Current molecular-genetic methods, such as SP-PCR, RP-PCR, *Aci*I digestion of SP-PCR products, pyrosequencing, or targeted NGS bisulfite sequencing analyze individual features of the expanded *DMPK* locus and/or give limited information about a certain feature. In addition, many studies are focused only on genetic variability of *DMPK* locus. Recently, it has been demonstrated that sequencing of native *FMR1* and *C9orf72* expanded loci from patient samples is feasible by long-read Oxford Nanopore technology and gives precise information on expansion size and DNA methylation pattern at single-molecule level [77]. In addition, sequencing of native *TCF4* expanded loci (associated with Fuchs Endothelial Corneal Dystrophy) by PacBio technology, another long-read sequencing technology, showed that identification of repeat interruptions is also possible [78]. Due to the constant improvement of long-read sequencing technologies and their ever-increasing availability, it can be anticipated that expansion size, level of somatic instability, pattern of variant repeats across the whole expansions, and pattern and level of DNA methylation will be obtained as readouts from a single analysis. This new methodological approach will lead to a more complete picture of genetic and epigenetic variability of *DMPK* locus in DM1 patients.

Furthermore, in many studies analyzing patients with variant repeats, only genetic, but not clinical description of patients is available or clinical description is insufficient to draw any conclusion about contribution of repeat interruptions to DM1 phenotype variability. Additional limitation is that comparison between patients with vs. without variant repeats should be controlled, at least, for the age at sampling and the expansion size. In two studies, this problem was partially resolved [48,52]. However, the problem of a small number of patients included in the studies and a low statistical power persists. A promising approach is to use mathematical modeling of a clinical symptom of interest obtained from a large referent cohort that takes into account the contribution of individual-specific factors. Such an approach was shown to be valuable for getting the first insight into the modifying role of variant repeats on age at disease onset [56]. Since DM1 is a rare disorder where variant repeats are detected in only 3–5% of patients, there is a need for international, multicentric collaboration to resolve which DM1 symptoms are modified by the variant repeats and to what extent. In addition, studies on patient tissues other than blood, and studies on the roles of variant repeats in the molecular pathogenesis of DM1 are also needed.

DM1 is an incurable disease and big hopes and efforts are oriented toward developing new therapies or repurposing drugs that target the genetic cause of the disease. Therapeutic approaches are designed to target either the *DMPK* expansion itself, including CRISPR/Cas9 gene editing strategies [79], or its downstream effects, such as RNA toxicity, including antisense oligonucleotide approach [80] and signaling pathways [81]. An additional therapy approach that acts on the DNA level is targeting expansion instability in somatic cells and is being developed for repeat expansion diseases (reviewed in [63]). Results of studies on somatic instability and age at disease onset in DM1 patients with variant repeats [36,56] and a longitudinal study in DM1 patients with pure repeats [19] support this new therapeutic approach because they show that variation in somatic instability contributes to variation in age at disease onset.

In conclusion, the discovery of variant repeats has opened a new perspective on structural variability of *DMPK* expansion that impacts epigenetic variability as well. An immediate implication of clinical relevance is the need for performing genetic diagnostics by RP-PCR which targets both ends of *DMPK* expansion and should currently be the standard procedure in diagnostic laboratories. Another important lesson learned is a need for a more complete picture of the interplay between genetic, epigenetic, and phenotype variability in DM1. Harnessing long-read sequencing technologies and their ability to sequence native DNA in order to obtain all relevant genetic and epigenetic parameters of the *DMPK* expanded locus, accompanied by studies on large patient groups, is necessary to accumulate data that will complement and, likely, direct efforts in developing therapies that target the genetic cause of the disease.

## Data Availability

Not applicable.
